# Draft Genome Sequence of a *Pseudomonas aeruginosa* Strain Able To Decompose *N*,*N*-Dimethyl Formamide

**DOI:** 10.1128/genomeA.01609-15

**Published:** 2016-02-04

**Authors:** Lingyue Yan, Ming Yan, Lin Xu, Li Wei, Liting Zhang

**Affiliations:** a Bioinformatics and Genetic Engineering Biology, Nanjing Tech University, Nanjing, China; b CapitalBio Technology, Beijing, China

## Abstract

*Pseudomonas aeruginosa* is a Gram-negative bacterium, which uses a variety of organic chemicals as carbon sources. Here, we report the genome sequence of the Cu1510 isolate from wastewater containing a high concentration of *N*,*N*-dimethyl formamide.

## GENOME ANNOUNCEMENT

*Pseudomonas aeruginosa*, which is infectious to plants (1, 2) and animals (3, 4), including human beings (5–7), is widely studied for its multidrug resistance (8–11). It is also used to degrade organic chemicals in wastewater because it can utilize a large range of organic chemicals as carbon sources (12–14) and survive in the presence of multiple metal ions (15–18). *N*,*N*-dimethyl formamide, an industrial organic solvent, possesses hepatotoxicity, embryotoxicity, teratogenicity, and possible carcinogenicity (19), but it is difficult to degrade. Here, we present the draft genome sequence of a wild-type strain of *P. aeruginosa* capable of degrading *N*,*N*-dimethyl formamide.

The Cu1510 isolate was selected from wastewater with M9-*N*,*N*-dimethyl formamide (DMF) medium containing 10% *N*,*N*-dimethyl formamide (the only carbon source) and then cultivated in the LB medium at 37°C overnight. Genomic DNA of Cu1510 was extracted according to the instructions of a Biomiga bacterial genomic DNA (gDNA) kit (product no. GD2411-01). The quality and quantity of DNA were assayed using a K5500 microspectrophotometer (Kaiao) and agarose gel electrophoresis. Genome sequencing was performed at CapitalBio Technology using Illumina technology with a DNA sequencing (DNA-seq) paired-end protocol. A library was prepared with the NEBNext Ultra DNA library prep kit for Illumina, and the instrument used for sequencing was HiSeq 2500. The resulted 15 million reads were assembled with Velvet 1.1 (20) and then refined with SEQuel version 1.0.2 (21). The remaining contigs were submitted to the ProDeGe website (22) for automatic decontamination. e-RGA (23) was applied for genome mapping. The resulting 6,122,906-bp draft genome containing 5,336 genes is rich in G+C content (66.65%). Finally, the assembled sequence was submitted to the National Center for Biotechnology Information (NCBI) Prokaryotic Genome Annotation Pipeline (PGAP) (24) for annotation. A detailed analysis on the *P. aeruginosa* pathway of DMF biodegradation will be presented in a later essay.

### Nucleotide sequence accession numbers.

The genome project was submitted to DDBJ/EMBL/GenBank under the accession no. CP013144. The version described in this paper is the first version, CP013144.1.

## References

[1] Kumar A, Munder A, Aravind R, Eapen S, Tümmler B, Raaijmakers JM 2012 Friend or foe: genetic and functional characterization of plant endophytic *Pseudomonas aeruginosa*. Environ Microbiol 15:764–779. doi:10.1111/1462-2920.12031. 23171326

[2] Hariprasad P, Chandrashekar S, Brijesh SS, Niranjana SR 2014 Mechanisms of plant growth promotion and disease suppression by *Pseudomonas aeruginosa* strain 2apa. J Basic Microbiol 54:792–801. doi:10.1002/jobm.201200491. 23681707 10.1002/jobm.201200491

[3] Paul W, Reena V, Bonnell JC, DiGiandomenico A, Camara M, Cook K, Peng L, Zha J, Chowdury, Sellman B, Stover CK 2014 A novel anti-PcrV antibody providing enhanced protection against *Pseudomonas aeruginosa* in multiple animal infection models.. Antimicrob Agents Chemother 58:4384–4391. doi:10.1128/AAC.02643-14. 24841258 PMC4136058

[4] Pang Y, Zhang L, Zhou S, Zhoua S, Yama KL, Liub L, Sheen S 2015 Growth behavior prediction of fresh catfish fillet with *Pseudomonas aeruginosa* under stresses of allyl isothiocyanate, temperature and modified atmosphere. Food Control 47:326–333. doi:10.1016/j.foodcont.2014.07.030.

[5] Li Y, Zhang X, Wang C, Hu Y, Niu X, Pei D, He Z, Bi Y 2015 Characterization by phenotypic and genotypic methods of metallo-[beta]-lactamase-producing *Pseudomonas aeruginosa* isolated from patients with cystic fibrosis. Mol Med Rep 11:494. doi:10.3892/mmr.2014.2685. 25323940

[6] Müller FM, Bend J, Huttegger I, Möller A, Schwarz C, Abele-Horn M, Ballmann M, Bargon J, Baumann I, Bremer W, Bruns R, Brunsmann F, Fischer R, Geidel C, Hebestreit H, Hirche TO, Hogardt M, Illing S, Koitschev A, Kohlhäufl M 2015 S3 guidelines on pulmonary disease in cystic fibrosis; module 1: diagnostics and therapy after initial detection of *Pseudomonas aeruginosa*/S3-leitlinie “Lungenerkrankung bei mukoviszidose”; modul 1: diagnostik und therapie nach dem ersten nachweis von *Pseudomonas aeruginosa*. Monatsschr Kinderheilkd 163:590–599. (In German.) doi:10.1007/s00112-015-3354-3.

[7] Johansen HK, Gøtzsche PC 2008 Vaccines for preventing infection with *Pseudomonas aeruginosa* in cystic fibrosis.. Cochrane Database Syst Rev (6):CD001399. doi:10.1002/14651858.CD001399.pub2. 10.1002/14651858.CD001399.pub2 18843616

[8] Gilani M, Munir T, Latif M, Rehman S, Ansari M, Hafeez A, Najeeb S, Saad N, Gilani M 2015 *In vitro* efficacy of doripenem against *Pseudomonas aeruginosa* and *Acinetobacter baumannii* by E-test. J Coll Physicians Surg Pak 25:726–729. doi:10.2015/JCPSP.726729. 26454387

[9] Xu Z, Gao Y, He J, Xu W, Jiang M, Jin H 2015 Effects of azithromycin on *Pseudomonas aeruginosa* isolates from catheter-associated urinary tract infection. Exp Ther Med 9:569. doi:10.3892/etm.2014.2120. 25574236 PMC4280936

[10] Oliver A, Mulet X, López-Causapé C, Juan C 2015 The increasing threat of *Pseudomonas aeruginosa* high-risk clones. Drug Resist Update 21–22:41–59. doi:10.1016/j.drup.2015.08.002. 26304792

[11] Cirioni O, Ghiselli R, Silvestri C, Minardi D, Gabrielli E, Orlando F, Rimini M, Brescini L, Muzzonigro G, Guerrieri M, Giacometti A 2011 Effect of the combination of clarithromycin and amikacin on *Pseudomonas aeruginosa* biofilm in an animal model of ureteral stent infection. J Antimicrob Chemother 66:1318–1323. doi:10.1093/jac/dkr107. 21406436

[12] Kumari S, Chetty D, Ramdhani N, Bux F 2013 Phenol degrading ability of *Rhodococcus pyrinidivorans* and *Pseudomonas aeruginosa* isolated from activated sludge plants in South Africa. J Environ Sci Health 48:947–953. doi:10.1080/10934529.2013.762740. 23485246

[13] Hassanshahian M, Ahmadinejad M, Tebyanian H, Kariminik A 2013 Isolation and characterization of alkane degrading bacteria from petroleum reservoir waste water in Iran (Kerman and Tehran provenances). Mar Pollut Bull 73:300–305. doi:10.1016/j.marpolbul.2013.05.002. 23790464

[14] Choudhary S, Sar P 2011 Uranium biomineralization by a metal resistant *Pseudomonas aeruginosa* strain isolated from contaminated mine waste.. J Hazard Mater 186:336–343. doi:10.1016/j.jhazmat.2010.11.004. 21112694

[15] Cornelis P 2010 Iron uptake and metabolism in pseudomonads. Appl Microbiol Biotechnol 86:1637–1645. doi:10.1007/s00253-010-2550-2. 20352420

[16] Ochsner UA, Wilderman PJ, Vasil AI, Vasil ML 2002 GeneChip expression analysis of the iron starvation response in *Pseudomonas aeruginosa*: identification of novel pyoverdine biosynthesis genes. Mol Microbiol 45:1277–1287. doi:10.1046/j.1365-2958.2002.03084.x. 12207696

[17] Paraneeiswaran A, Shukla SK, Rao TS, Prashanth K 2014 Removal of toxic Co-EDTA complex by a halophilic solar-salt-pan isolate *Pseudomonas aeruginosa* SPB-1. Chemosphere 95:503–510. doi:10.1016/j.chemosphere.2013.09.107. 24183626

[18] Yin NH, Sivry Y, Avril C, Borensztajn S, Labanowski J, Malavergne V, Lens PNL, Rossano S, Hullebusch EDV 2014 Bioweathering of lead blast furnace metallurgical slags by *Pseudomonas aeruginosa*. Int Biodeterior Biodegrad 86:372–381. doi:10.1016/j.ibiod.2013.10.013.

[19] Dar-Bin S, Chia-Chun CC, Tung-Sheng S, Huo-Mu T, Yau-Hui W, Ho-Yuan C 2007 Mitochondrial D*N*A alterations in blood of the humans exposed to *N*,*N*-dimethylformamide.. Chem Biol Interact 165:211–219. doi:10.1016/j.cbi.2006.12.008. 17254560

[20] Zerbino DR, Birney E 2008 Velvet: algorithms for *de novo* short read assembly using de Bruijn graphs. Genome Res 18:821–829. doi:10.1101/gr.074492.107. 18349386 PMC2336801

[21] Ronen R, Boucher C, Chitsaz H, Pevzne P 2012 SEQuel: improving the accuracy of genome assemblies. Bioinformatics 28:i188–i196. doi:10.1093/bioinformatics/bts219. 22689760 PMC3371851

[22] Tennessen K, Andersen E, Clingenpeel S, Rinke C, Lundberg DS, Han J, Dangl JL, Ivanova N, Woyke T, Kyrpides N 2015 ProDeGe: a computational protocol for fully automated decontamination of genomes. ISME J 10:269–272. doi:10.1038/ismej.2015.100. 26057843 PMC4681846

[23] Vezzi F, Cattonaro F, Policriti A 2011 E-RGA: enhanced Reference Guided Assembly of Complex genomes. EMBnet J 17. doi:10.14806/ej.17.1.208.

[24] Tatusova T, DiCuccio M, Badretdin A, Chetvernin V, Ciufo S, Li W 2013 Prokaryotic genome annotation pipeline. The NCBI handbook, 2nd ed. National Center for Biotechnology Information, Bethesda, MD.

